# Clinical Efficacy and Safety of Intramuscular Injections of Autologous Total IgG in Patients With Chronic Spontaneous Urticaria: An Open‐Label Prospective Pilot Trial

**DOI:** 10.1111/exd.70249

**Published:** 2026-04-13

**Authors:** Young‐Min Ye, Myung‐Eun Kim, Byul Kwon, Dong‐Ho Nahm

**Affiliations:** ^1^ Department of Allergy and Clinical Immunology Ajou University School of Medicine Suwon Korea

**Keywords:** autologous immunoglobulin, chronic spontaneous urticaria, immunoglobulin G, immunomodulatory therapy, urticaria activity score

## Abstract

Chronic spontaneous urticaria (CSU) remains challenging to manage in patients who do not respond adequately to antihistamines or currently available immunomodulatory therapies. Intramuscular injection of autologous total IgG (autologous immunoglobulin therapy: AIGT) has demonstrated clinical efficacy, safety and immunomodulatory effects in patients with moderate‐to‐severe atopic dermatitis in a randomized placebo‐controlled clinical trial. However, the clinical usefulness of AIGT in patients with CSU has not been evaluated. We conducted a prospective open‐label pilot study to assess the efficacy and safety of AIGT in antihistamine‐refractory CSU. Fifteen adults with CSU received nine weekly intramuscular injections of 100 mg autologous IgG from Week 0 through Week 8 (inclusive). The primary outcome was the change in Urticaria Activity Score over 7 days (UAS7) at Week 12 from baseline. Secondary outcomes included the Urticaria Control Test (UCT), chronic urticaria‐specific quality of life (CU‐QoL) scores and patient‐reported disease burden using a visual analogue scale (VAS). The median change in UAS7 at Week 12 from baseline was −13.0 (*p* < 0.001). Significant improvements in UCT, CU‐QoL and VAS were also observed at Week 12 from baseline (*p* < 0.05). In longitudinal analyses, improvements in symptom burden and quality of life were detectable from Week 4 and were maintained through Week 24. Serum total IgG increased by a median of +68.0 mg/dL from baseline to Week 12 (*p* < 0.05). No serious adverse events occurred. In conclusion, AIGT improved disease activity, urticaria control, quality of life and patient‐reported burden in patients with antihistamine‐refractory CSU. Further studies are needed to evaluate the clinical usefulness of AIGT in CSU.

## 1 Background

Chronic spontaneous urticaria (CSU) is a mast‐cell‐driven inflammatory skin disorder characterized by recurrent wheals and pruritus persisting for > 6 weeks [1]. Second‐generation H1‐antihistamines constitute the standard first‐line therapy, yet up to 40% of patients remain symptomatic despite dose escalation [2]. Omalizumab and cyclosporine are recommended for antihistamine‐refractory CSU; however, treatment responses vary considerably, and many patients fail to achieve durable disease control [3, 4]. This clinical heterogeneity highlights the need for additional therapeutic strategies targeting distinct underlying mechanisms.

The understanding of CSU has shifted towards recognizing the disease as a heterogeneous autoimmune and immune‐dysregulated condition [5, 6]. Evidence supports the presence of IgE autoantibodies (Type I autoimmunity) and IgG autoantibodies (Type IIb autoimmunity) directed against shared autoantigens, including FcɛRI and thyroid peroxidase [7, 8]. Dysregulated antigen processing, aberrant MHC class II‐mediated T‐cell activation and amplification of autoreactive pathways may further contribute to disease persistence [9]. Reports of CSU flares following COVID‐19 vaccination reinforce the concept of a primed immune dysregulation in which external immune stimuli amplify autoreactivity [10, 11].

Intramuscular injection of autologous total IgG (autologous immunoglobulin therapy: AIGT) has demonstrated clinical efficacy, safety and immunomodulatory effects in patients with moderate‐to‐severe atopic dermatitis by a randomized double‐blind placebo‐controlled clinical trial [12]. However, clinical efficacy and safety of AIGT in patients with CSU have not been evaluated.

## 2 Questions Addressed

- Does intramuscular injection of autologous total IgG (AIGT) improve urticaria activity and patient‐reported outcomes in patients with antihistamine‐refractory CSU?
- Are clinical benefits of AIGT sustained beyond the treatment period?
- Is AIGT safe and well tolerated?
- Does AIGT demonstrate immunomodulatory effects reflected in serum IgG and IgE concentrations?

## 3 Experimental Design

### 3.1 Study Population

This open‐label, prospective pilot trial enrolled 15 patients with CSU unresponsive to H1‐antihistamines and, in some cases, to anti‐IgE therapy or cyclosporine (Table 1). All participants maintained their baseline H1‐antihistamine regimen at a stable dose. Use of an additional licensed H1‐antihistamine was permitted as needed. Eligible patients aged 19–75 years had CSU for ≥ 6 months, persistent itchy wheals for ≥ 8 weeks despite treatment and a baseline UAS7 ≥ 16.

**TABLE 1:** Baseline clinical characteristics of the study subjects.

Characteristics	Median (IQR) or *n* (%)
Age (year)	49 (35–62)
Female sex, *n* (%)	12 (80.0%)
Duration of disease (year)	2.8 (1.5–4.0)
UAS7 (0–42)	25 (21–32)
UCT (0–16)	7 (4–8)
CU‐QoL (0–100)	54.4 (27.9–72.1)
Patient‐perceived burden (VAS, 0–10)	7.7 (5.9–9.5)
Concomitant atopic diseases, *n* (%)	
Asthma	1 (6.7%)
Allergic rhinitis	7 (46.7%)
Prior treatment before enrollment, *n* (%)	
Omalizumab	4 (26.7%)
Cyclosporine	3 (20.0%)
Methotreaxate	1 (6.7%)
Oral corticosteroid	2 (13.3%)
WBC (/μL)	5100 (4600–7000)
D‐dimer (μg/mL)	0.45 (0.36–1.17)
LDH (U/L)	171 (151–202)
Total IgE (kU/L, 0~114)	75 (21–204)
IgG (mg/dL, 916~1796)	1108 (1039–1254)
IgA (mg/dL, 93~365)	186 (169–234)
IgM (mg/dL, 40~260)	94 (51–170)
ASST positivity (%)	4 (26.7%)

*Note:* Data are presented as number (%) or medians (interquartile range).

Abbreviations: ASST, autologous serum skin test; CU‐QoL, Chronic Urticaria Quality of Life Questionnaire; UAS7, Urticaria Activity Score over 7 days; UCT, Urticaria Control Test; VAS, patient‐reported disease burden using a visual analogue scale.

### 3.2 Intervention

Autologous venous blood (400 mL) was collected from each participant using a double blood bag containing anticoagulant (Green Cross PBM, Seoul, Korea) at the initial screening visit (Week−4). Autologous total IgG (purity ≥ 97%) was purified from each participant's plasma using Protein A affinity chromatography [12]. A total of 100 mg of autologous total IgG was intramuscularly injected in each patient (50 mg per gluteal muscle) once weekly from Week 0 through Week 8 (inclusive), resulting in nine injections with a cumulative dose of 900 mg.

### 3.3 Outcomes

The primary endpoint was the change in Urticaria Activity Score over 7 days (UAS7) from baseline to Week 12. Secondary endpoints included Urticaria Control Test (UCT), chronic urticaria‐specific quality of life (CU‐QoL), and patient‐reported disease burden using a visual analogue scale (VAS) from baseline to Week 12. Longitudinal clinical assessments of UAS7, UCT, CU‐QoL and VAS were conducted at Weeks 4, 8, 12, 16, 20 and 24 to evaluate time‐dependent trends.

Responder analyses were prespecified using clinically relevant thresholds, including proportions of participants achieving UAS7 ≤ 6 (minimal disease activity), UAS7 = 0 (complete remission) and UCT ≥ 12 (well‐controlled) at each follow‐up visit.

Exploratory subgroup analyses compared responder rates and changes in clinical and immunologic parameters according to baseline ASST (autologous serum skin test) status and according to UCT response at Week 12. ASST was performed at baseline and Week 12. Serum levels of total IgE were measured using the ImmunoCAP assay (Thermo Fisher Scientific, Waltham, MA, USA). Serum concentrations of IgG, IgA and IgM were assayed by turbidimetric immunoassay using a COBAS Integra analyzer (F. Hoffmann‐La Roche, Basel, Switzerland). Serum concentrations of IgG subclasses 1, 2, 3 and 4 were measured by turbidimetric immunoassay using the Optilite analyzer (Binding Site, Birmingham, U.K.). Laboratory safety parameters were measured at baseline and Week 12.

### 3.4 Ethics

The study was approved by the institutional review board (MED‐INT‐21‐320) and registered with the Clinical Research Information Service of Korea (KCT0006814). All participants provided written informed consent.

### 3.5 Statistical Analysis

The primary endpoint (change in UAS7 from baseline to Week 12) was analysed using the Wilcoxon signed‐rank test. Secondary continuous outcomes at Week 12 (UCT, CU‐QoL and VAS) were analysed using the same approach.

To evaluate longitudinal trends across Weeks 4, 8, 12, 16, 20 and 24, generalized estimating equations (GEE) adjusted for age and sex were applied to continuous variables, accounting for within‐subject correlation across repeated measurements.

Responder outcomes (proportions achieving UAS7 ≤ 6, UAS7 = 0 and UCT ≥ 12) were analysed using exact McNemar tests comparing each follow‐up time point with baseline. Missing follow‐up data for responder analyses were handled using a non‐responder imputation approach. All tests were two‐sided, and *p* < 0.05 was considered statistically significant.

## 4 Results

### 4.1 Baseline Characteristics

Fifteen patients completed the study through Week 12 (median age 49 years; 80% female). At baseline, disease activity was in the moderate‐to‐severe range, with a median UAS7 of 25 (interquartile range [IQR] 21–32), indicating substantial symptom burden. Disease control was generally poor, as reflected by a median UCT score of 7 (IQR 4–8), and CU‐specific quality of life was markedly impaired (54.4, IQR 27.9–72.1). The median VAS score for perceived disease burden was 7.7 (5.9–9.5), consistent with a high subjective impact of CSU on daily life. Comorbid allergic rhinitis was reported in 46.7% (Table 1).

Six participants had previously received omalizumab or immunosuppressive agents without achieving adequate disease control, whereas the remaining patients had been treated with H1‐antihistamines alone (Table S1). Baseline UAS7 values were comparable between these subgroups, indicating that prior exposure to biologics or immunosuppressants did not simply reflect more severe disease at study entry. In line with this, CU‐QoL scores were significantly correlated with both UAS7 (r = −0.627, *p* = 0.012) and VAS (r = −0.583, *p* = 0.023), underscoring the close relationship between objective disease activity and patient‐perceived burden in this cohort.

### 4.2 Clinical Outcomes at Week 12

**FIGURE 1:**
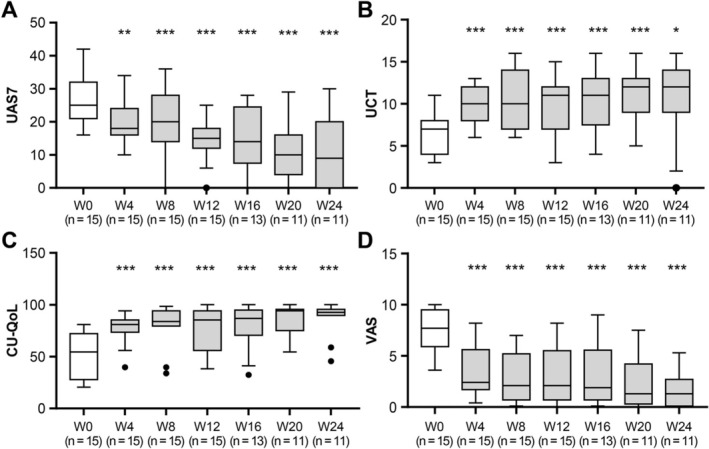
Longitudinal changes in UAS7 (A), UCT (B), CU‐QoL (C) and VAS (D) from baseline to Weeks 4, 8, 12, 16, 20 and 24. Data are presented as median and interquartile range (IQR). Within‐subject time‐dependent changes from baseline were analysed using generalized estimating equations adjusted for age and sex. ***, *p* < 0.001; **, *p* < 0.01; *, *p* < 0.05; ns, not significant.

AIGT significantly improved urticaria activity and patient‐reported outcomes at Week 12 from baseline (Figure 1). For the primary endpoint, the median change in UAS7 from baseline to Week 12 was −13.0 (IQR −17.0 to −5.0, *p* < 0.001). In parallel, the UCT score increased by a median of +4.0 points (IQR 1.0 to 5.0, *p* = 0.003), indicating a clinically meaningful gain in disease control. CU‐QoL scores increased by a median of +23.6 (IQR 16.2 to 36.8, *p* = 0.001), and VAS scores decreased by −4.3 (IQR −6.8 to −1.3, *p* = 0.001), indicating clinically meaningful improvement in disease activity, disease control and patient‐perceived burden.

At Week 12, 13.3% of participants achieved UAS7 ≤ 6, 6.7% achieved complete remission (UAS7 = 0); however, these categorical improvements did not reach statistical significance. In contrast, the proportion achieving UCT ≥ 12 increased significantly beginning at Week 8 (46.7%) and remained elevated at Week 12 (40.0%), reflecting well‐controlled disease (Table 2).

**TABLE 2:** Longitudinal changes in clinical outcomes and responder rates during the study period.

	Week 0 (*n* = 15)	Week 4 (*n* = 15)	Week 8 (*n* = 15)	Week 12 (*n* = 15)	Week 16 (*n* = 13)	Week 20 (*n* = 11)	Week 24 (*n* = 11)
UAS7 (0–42)	25 (21, 32)	18 (16, 24)**	19 (7, 22)***	15 (12, 18)***	14 (9, 24)***	10 (4, 16)***	9 (0, 20)***
UAS7 ≤ 6 (%)^a^	0	0	3 (20.0)	2 (13.3)	3 (20.0)	5 (33.3)	4 (26.7)
UAS7 = 0 (%)^a^	0	0	1 (6.7)	1 (6.7)	2 (13.3)	2 (13.3)	3 (20.0)
UCT (0–16)	7 (4, 8)	10 (8, 12)***	11 (9, 13)***	11 (7, 12)***	11 (8, 13)***	12 (9, 13)***	12 (9, 14)*
UCT ≥ 12 (%)^a^	0	4 (26.7)	7 (46.7)*	6 (40.0)*	6 (40.0)*	8 (53.3)**	6 (40.0)*
VAS	7.7 (5.9, 9.5)	2.4 (1.7, 5.6)***	2.1 (0.7, 5.2)***	2.1 (0.7, 5.5)***	1.9 (0.9, 4.9)***	1.3 (0.3, 4.2)***	1.3 (0.1, 2.7)***
CU‐QoL	54.4 (27.9, 72.1)	80.9 (73.5, 85.3)***	83.8 (79.4, 94.1)***	85.3 (55.9, 94.1)***	86.8 (80.9, 94.1)***	94.1 (75.0, 95.6)***	92.6 (89.7, 95.6)***

*Note:* Data are presented as median (interquartile range). *p < 0.05, **p < 0.01, ***p < 0.001 compared with baseline (Week 0). Continuous variables were analysed using generalized estimating equations adjusted for age and sex, and categorical variables were analysed using exact McNemar tests.

Abbreviations: CU‐QoL, chronic urticaria‐specific quality of life; UAS7, Urticaria Activity Score over 7 days; UCT, Urticaria Control Test; VAS, visual analogue scale for patient‐perceived disease burden.

^a^ Missing follow‐up data were imputed as non‐response.

Exploratory analyses stratified by baseline ASST status did not show significant differences in changes in clinical outcomes or immunoglobulin levels from baseline to Week 12. Although numerically higher responder rates were observed among ASST‐positive participants, these differences did not reach statistical significance.

Importantly, these improvements were not confined to patients naïve to immunomodulatory therapy. Among the six patients with inadequate prior responses to omalizumab or cyclosporine, most also experienced reductions in UAS7 and VAS and increases in UCT and CU‐QoL at Week 12. These findings suggest that AIGT may provide benefit even in a treatment‐refractory population.

### 4.3 Sustained Effects to Week 24

After Week 12, two patients discontinued follow‐up because of insufficient symptomatic improvement, and two were lost to follow‐up after Week 16, leaving 11 participants who completed the Week 24 visit. Despite the reduced sample size, the overall pattern suggesting that clinical benefit persisted beyond the active treatment phase (Figure 1). Median UAS7 decreased from 25 (21–32) at baseline to 19 (7–22) at Week 8 (*p* = 0.019), 15 (12–18) at Week 12 (*p* = 0.003) and 14 (8–25) at Week 16 (*p* = 0.021). This improvement was sustained at Week 24, with a median UAS7 of 9 (IQR 0–20, *p* = 0.043 vs. baseline).

VAS scores showed a similar trajectory, decreasing from 8 (6–10) at baseline to 2 (1–5) at Week 8 (*p* = 0.005), 2 (1–6) at Week 12 (*p* = 0.007), 2 (1–6) at Week 16 (*p* = 0.021) and 1 (0–3) at Week 24 (*p* = 0.013). UCT and CU‐QoL also improved significantly at weeks 8 and 16 compared with baseline, median UCT 11 (IQR 9–13, *p* = 0.004) and 11 (8–13, *p* = 0.020), median CU‐QoL 84 (IQR 79–94, *p* = 0.003) and 87 (71–95, *p* = 0.006), respectively. Although these improvements remained numerically favourable at Week 24 (UCT 12 [9, 10, 11, 12, 13, 14], CU‐QoL 93 [90‐96]), statistical significance was not reached, most likely owing to limited sample size at this time point. Notably, three of 11 patients (27.3%) achieved complete remission (UAS7 = 0) at Week 24 (Table S1).

In longitudinal analyses using generalized estimated equations adjusted for age and sex, all continuous outcome measures (UAS7, UCT, VAS and CU‐QoL) showed significant improvements from baseline beginning at Week 4, and these time‐dependent differences were maintained through Week 24 (Table 2). The proportion of participants achieving UCT ≥ 12 increased significantly from Week 8 through Week 24. In contrast, although the proportions achieving UAS7 = 0 or UAS7 ≤ 6 increased numerically over time, these changes did not reach statistical significance during the study period (Table 2). Responder rates were calculated using a non‐responder imputation approach for missing follow‐up data.

### 4.4 Changes in Serum Concentrations of Immunoglobulins and Skin Reactivity to Autologous Serum

Changes in serum immunoglobulin concentrations and ASST reactivity were evaluated as exploratory assessments of potential biologic plausibility (Table 3). Serum total IgE levels did not change significantly from baseline to Week 12 (median change +2.0 kU/L, *p* = 0.808). In contrast, serum total IgG concentrations increased significantly, with a median increase of +68.0 mg/dL (*p* = 0.048). Among IgG subclasses, modest but statistically significant increases were observed in IgG1 (*p* = 0.041) and IgG2 (*p* = 0.022), whereas IgG3 and IgG4 levels did not change significantly after AIGT treatment. No significant changes were detected in serum IgA and IgM concentrations (*p* > 0.05). The proportion of patients with a positive ASST decreased from 26.7% (4/15) at baseline to 6.7% (1/15) at Week 12; however, this reduction did not reach statistical significance.

**TABLE 3:** Changes in serum immunoglobulin concentrations and ASST positivity from baseline to Week 12.

	Week 0	Week 12	*p*
Total IgE, kU/L	75 (21, 204)	92 (21, 118)	0.808
IgG, mg/dL	1108 (1039, 1254)	1205 (1090, 1402)	0.048
IgG1, mg/dL	536.3 (469.1, 675.0)	556.8 (508.3, 646.5)	0.041
IgG2, mg/dL	457.1 (371.8, 491.5)	475.6 (370.9, 551.3)	0.022
IgG3, mg/dL	28.1 (17.9, 52.8)	29.6 (20.2, 56.5)	0.772
IgG4, mg/dL	30.5 (20.8, 41.4)	31.6 (21.4, 40.4)	0.123
IgA, mg/dL	186 (169, 234)	199 (171, 253)	0.158
IgM, mg/dL	94 (51, 170)	96 (59, 172)	0.480
ASST (%)	4/15 (26.7)	1/15 (6.7)	0.125

*Note:* Data are expressed as medians (IQR; 25th to 75th percentiles). *p < 0.05, **p < 0.01, ***p < 0.001 compared with baseline (Week 0). Continuous variables were analysed using the Wilcoxon signed‐rank test, and categorical variables were analysed using the exact McNemar test.

Abbreviation: ASST, autologous serum skin test.

### 4.5 Safety

AIGT was generally well tolerated. No serious adverse events were observed during the study period. Six participants reported mild and transient symptoms, including upper respiratory infections, fatigue, headache and cystitis, none of which led to treatment discontinuation. Haematological (complete blood count with differential counts of leukocytes) and biochemical laboratory parameters (routine chemistry profiles) remained within clinically acceptable ranges, and no consistent trends suggesting organ toxicity were identified.

During the first 12 weeks, two participants required rescue antihistamines. From Week 16 through the end of follow‐up, five participants used rescue antihistamines, with individual use ranging from 1 to 28 doses.

## 5 Conclusions & Perspectives

This prospective pilot trial provides preliminary clinical evidence that AIGT could be a safe and potentially beneficial therapeutic option for patients with antihistamine‐refractory CSU. Statistically significant improvements in UAS7, UCT, CU‐QoL and VAS were observed at Week 12 and were sustained through Week 24 in longitudinal analyses. Importantly, several patients with inadequate prior responses to omalizumab or cyclosporine also demonstrated clinically meaningful improvement, suggesting potential utility in treatment‐refractory populations. Notably, improvements in symptom burden (UAS7 and VAS) and CU‐QoL were detectable as early as Week 4, whereas improvements in disease control (UCT ≥ 12) became statistically significant by Week 8 and were maintained through Week 24, suggesting a sustained and time‐dependent therapeutic effect.

This study could be historically considered as one part of continued efforts over more than 100 years to improve outcomes in patients with CSU by intramuscular injection of autologous blood or serum by many past physicians [13, 14, 15]. Intramuscular injection of autologous whole blood (autologous blood therapy or autohemotherapy) or serum (autologous serum therapy or autoserum therapy) has been explored for decades with the rationale of dampening autoreactive pathways. However, the heterogeneity of serum‐derived components and inconsistent clinical outcomes have limited mechanistic interpretation and widespread adoption [14, 16, 17, 18]. High‐dose intravenous immunoglobulin has shown benefit in selected patients with autoimmune CSU, particularly those with functional IgG autoantibodies, but its use is constrained by high cost, systemic exposure and infusion‐related risks [19, 20, 21].

In contrast, AIGT involves the administration of a small, purified and standardized dose of autologous total IgG, potentially allowing a more targeted engagement of idiotype‐anti‐idiotype networks while minimizing nonspecific serum components. The modest but statistically significant increase in serum concentration of total IgG, IgG1, and IgG2 subclasses from baseline to Week 12 observed in this study supports biologic plausibility. However, a direct mechanistic linkage between IgG changes and clinical response was not established. In exploratory analyses, changes in total IgE, total IgG and IgG subclasses did not differ significantly according to baseline ASST status or UCT response at Week 12. Although the proportion of ASST positivity decreased following AIGT treatment, this reduction did not reach statistical significance. These findings suggest that while AIGT may influence circulating immunoglobulin levels, the clinical relevance of these changes and their relationship to autoimmune endotypes remain to be clarified in larger controlled studies.

The persistence of clinical improvement through Week 24 following nine weekly intramuscular injections administered from Week 0 through Week 8 (inclusive) suggests that AIGT may exert effects extending beyond transient symptomatic relief. Further studies are needed to clarify the immunomodulatory mechanisms of AIGT including activation of anti‐idiotypic regulatory T cells, neutralization of pathogenic antibodies, modulation of mast‐cell‐activation thresholds [12, 19, 22, 23, 24].

Nevertheless, given the small sample size, open‐label design and lack of a control group, these findings should be interpreted with caution. Further studies with a larger sample size and randomized controlled design incorporating mechanistic endpoints, such as changes in autoreactive IgE/IgG, mast‐cell‐activation signatures and antigen presenting cell phenotypes, are needed to validate clinical efficacy and clarify the mechanism of action of AIGT in CSU.

In conclusion, AIGT might provide an additional alternative therapeutic option for patients with CSU. However, further studies are needed to evaluate the clinical usefulness of AIGT for patients with CSU.

## Author Contributions

D.‐H.N.: conceptualization, funding acquisition, investigation, supervision, writing – review and editing. B.K. and M.‐E.K.: methodology, data curation, project administration. Y.‐M.Y.: investigation, formal analysis and writing – original draft. All authors reviewed and approved the final version of the manuscript.

## Funding

This work was supported by the National Research Foundation of Korea, NRF‐2022R1A2C2006607; the GRRC program of Gyeonggi Province, GRRCAjou2023‐B01.

## Conflicts of Interest

The authors have no conflicts of interest to declare related to this study, except that Dr. Dong‐Ho Nahm has served as a consultant for Autobulin Therapeutics.

## Supporting information


**Table S1:** Summary of prior treatments before screening and UAS7 at Weeks 0 and 12.

## Data Availability

The data that support the findings of this study are available on request from the corresponding author. The data are not publicly available due to privacy or ethical restrictions.
