# Supplementary material for: Clinical Efficacy and Safety of Intramuscular Injections of Autologous Total IgG in Patients With Chronic Spontaneous Urticaria: An Open‐Label Prospective Pilot Trial
Source: Exp Dermatol. 2026 Apr 13;35(4):e70249. doi: 10.1111/exd.70249 (PMC13071865; doi:10.1111/exd.70249)
Supplement: Supplementary file 1 — Table S1: Summary of prior treatments before screening and UAS7 at Weeks 0 and 12. [file EXD-35-e70249-s001.docx]

Supplementary Table S1. Summary of prior treatments before screening and UAS7 at weeks 0 and 12

| Subject No | Sex/Age | Prior Medication | Relative  Start-End | UAS7  at  W0 | UAS7  at W12 | UAS7 at W24 |
| --- | --- | --- | --- | --- | --- | --- |
| 1 | F/45 | 2 H1AH | W-15 ~ W-4 | 30 | 12 | 14 |
| 2 | F/45 | 2 H1AH | W-12 ~ W-4 | 23 | 18 | NA |
| 3 | F/31 | 2 H1AH | W-51 ~ W-4 | 23 | 10 | 9 |
| 4 | F/25 | 2 H1AH | W-12 ~ W-4 | 35 | 16 | 20 |
| 5 | F/50 | 3 H1AH | W-28 ~ W-4 | 21 | 19 | 30 |
| 6 | F/69 | 4 H1AH | W-26 ~ W-4 | 35 | 18 | 6 |
| 7 | F/57 | 1 H1AH | W-5 ~ W-4 | 16 | 0 | 0 |
| 8 | F/62 | 4 H1AH | W-11 ~ W-4 | 30 | 13 | 0 |
| 9 | M/35 | 1 H1AH | W-10 ~ W-4 | 25 | 14 | 7 |
| 10 | F/47 | 4 H1AH  Cyclosporine (100mg/d) | W-62 ~ W-4  W-56 ~ W-5 | 21 | 16 | NA |
| 11 | F/69 | 4 H1AH  Omalizumab (300mg)  Cyclosporine (100mg/d)  Prednisolone (10mg/d) | W-91 ~ W-4  W-91 ~ W-55  W-51 ~ W-14  W-14 ~ W-5 | 17 | 13 | NA |
| 12 | M/60 | 2 H1AH  Methotrexate (10mg/w) | W-13 ~ W-4  W-13 ~ W-5 | 18 | 17 | 18 |
| 13 | F/47 | 4 H1AH  Cyclosporine (100mg/d)  Omalizumab (300mg) | W-123 ~ W-4  W-118 ~ W-110  W-63 ~ W-12 | 32 | 25 | NA |
| 14 | F/68 | 3 H1AH  Cyclosporine (100mg/d)  Omalizumab (150mg)  Prednisolone (5mg/d) | W-138 ~ W-4  W-134 ~ W-123  W-123 ~ W-15  W-15 ~ W-7 | 42 | 6 | 0 |
| 15 | M/27 | 2 H1AH  Omalizumab (150mg) | W-631 ~ W-4  W-29 ~ W-13 | 28 | 15 | 28 |

AIGT, intramuscular injection of autologous total IgG; F, female; M, male; H1AH, H1-antihistamine; Relative start-end (week), relative to study date; UAS7, urticaria activity score over 7 days; W, week; NA, not applicable
